# Association of *COL9A3* trp3 polymorphism with intervertebral disk degeneration: a meta-analysis

**DOI:** 10.1186/s12891-018-2297-y

**Published:** 2018-10-20

**Authors:** Donghua Huang, Xiangyu Deng, Kaige Ma, Fashuai Wu, Deyao Shi, Hang Liang, Sheng Chen, Zengwu Shao

**Affiliations:** Department of Orthopaedics, Union Hospital, Tongji Medical College, Huazhong University of Science and Technology, Wuhan, 430022 China

**Keywords:** *COL9A3*, Single nucleotide polymorphism, trp3, Intervertebral disk degeneration, Meta-analysis

## Abstract

**Background:**

Intervertebral disk degeneration **(**IDD) is a common musculoskeletal disease associated with genetic factors. *COL9A3* gene encodes the α3 (IX) chain of type IX collagen that is part of the interior structure of the disc. Mutations in COL9A3 gene sequence, leading to an Arg103Trp substitution in its 3 chain (the Trp3 allele at rs61734651 site), respectively, have been found to be connected with IDD occurrence in several studies. However, those studies have showed conflict results. Thus, a meta-analysis has been performed to assess the associations between the *COL9A3* trp3 polymorphism and IDD.

**Methods:**

Data were gathered from the following four electronic databases: PubMed, Web of Science (WOS), Embase and Cochrane library up to January 01, 2018. The pooled odds ratio (polled ORs) and 95% confidence interval (CI) were calculated to evaluate the strength of relationship between the *COL9A3* trp3 polymorphism and IDD.

**Results:**

Eleven eligible studies with 1631 cases of IDD and 1366 controls were included in this meta-analysis. The results indicated that the *COL9A3* trp3 polymorphism was not associated with IDD (trp3 positive versus trp3 negative: OR = 1.31, 95%CI = 0.78–2.21, *P* = 0.309). Furthermore, the Egger’s test and the Begg funnel plot did not show any evidence of publication bias.

**Conclusions:**

Our results suggest that the *COL9A3* trp3 polymorphism might not be associated with IDD. Nor did we find any relationship in subgroup analyses stratified by gender and ethnicity. Future researches with larger samples are required to verify this outcome.

## Background

Low back pain (LBP), a common musculoskeletal disorder, involves the muscle, nerve, and bone tissues of back [1–3]. It has been reported that LBP ranked first in terms of disability and sixth in terms of total burden as part of the Global Burden of Disease 2010 Study [4]. Intervertebral disk degeneration (IDD), describing the natural destruction of intervertebral disk inside the spine, has been considered as one of the major causes to motor losses and LBP. The etiology and pathogenesis of IDD is so complicated that IDD is thought to be the results of co-effects of ageing and relevant environmental factors such as sporting activities, damage, occupation, and smoking [5–9]. However, there have been many articles establishing a close relationship between heredity and IDD recently [10–12].

For the last several years, many genes have been discovered to be associated with IDD, some of which are collagen genes, such as Collagen I, IX and XI genes [12–14]. Among these genes, the association of collagen type IX alpha 3 chain (*COL9A3)* gene polymorphism with IDD risk has been studied much frequently. *COL9A3* gene, located in the chromosome 20q13.3, encodes the α3 (IX) chain of type IX collagen which is part of the interior structure of the disc, nucleus pulposus [14–16]. Mutations in *COL9A3* gene leading to an Arg103Trp substitution in its 3 chain (the Trp3 allele at rs61734651 site), in other words, this change in collagen IX by substitution of glutamine by tryptophan, which is relative rare in collagen, can contribute to a disorder in the collagen properties of intervertebral disc. The increasing proportion of tryptophan in collagen can result in alterations in collagen triple helix, as well as interfering the interaction between collagens IX and II or disturbing the process of lysyl oxidase, which catalyzes cross-link formation, finally leading to disc disease [17–20].

However, recent studies have obtained conflicting results. Some of them, such as Toktas et al. [11] and Paassita et al. [17], found that trp3 gene was a risk fact of IDD or the spinal stenosis with spondylolisthesis which is one type of IDD. Others, such as Eskola et al. [21] and Rathod et al. [22], did not observe a relationship between trp3 and IDD. Besides, Bagheri et al. [18], only got an association of trp3 with IDD in males. A few articles reported the association of trp3 with ethnicity [23]. But no meta-analysis has investigated the association between IDD and *COL9A3* trp3 polymorphism up to now. Therefore, we performed a meta-analysis to evaluate the connection between them. In this study, we aim to identify the association of genetic mutations with IDD, which is likely to be of significant importance and might help identify ‘high-risk’ individuals of IDD or guide the clinical treatment of some specific individuals.

## Methods

### Strategy for literature search

In order to identify all articles that studied the association of *COL9A3* Trp3 polymorphism with IDD, we searched electronic databases including PubMed, Web of Science (WOS), Embase and Cochrane library up to January 01, 2018. The search strategy to screen out all possible articles involved the use of the following terms: (“COL9A3” OR “Collagen 9 alpha-3”) AND (“Gene polymorphism”) AND (“Intervertebral Disk Degeneration” OR “Disk Degeneration, Intervertebral” OR “IDD” OR “Disc Degeneration” OR “disc herniation” OR “low back pain”); (“Trp3” OR “rs61734651” OR “20q13.33” OR “arg103”) AND (“Gene polymorphism”) AND (“Intervertebral Disk Degeneration” OR “Disk Degeneration, Intervertebral” OR “IDD” OR “Disc Degeneration” OR “disc herniation” OR “low back pain”); (“COL9A3” OR “Collagen 9 alpha-3”) AND (“Trp3” OR “rs61734651” OR “20q13.33” OR “arg103”) AND (“Intervertebral Disk Degeneration” OR “Disk Degeneration, Intervertebral” OR “IDD” OR “Disc Degeneration” OR “disc herniation” OR “low back pain”). In order to increase the sensitivity of the searching strategy, both MeSH terms and free words were applied.

### Inclusion and exclusion criteria

Studies included in this meta-analysis should satisfy the following inclusion criteria: (1) Evaluation of the association between *COL9A3* trp3 polymorphism and the risk of IDD; (2) Human subjects; (3) Case-control study; and (4) Available genotype data were provided to calculate the odds ratios (ORs) and 95% confidence interval (CI).

Correspondingly, the exclusion criteria were defined as: (1) Comments, reviews or animal studies; (2) Duplicate reports with previous publications; (3) the study only described data of case population; (4) Studies without available genotype frequencies.

All retrieved articles were evaluated and discussed to achieve accordance by two junior investigators depending on the inclusion and exclusion strategies independently. If a conflict (among the basic information, data, and the quality of articles separately extracted by two investigators) still existed, a senior author was invited to extract the specific data independently using blind method. Then comparing the results with the two junior investigators to solve the problem and finally come to a consistency.

### Data extraction

The following characteristics of each study were collected: (1) name of the first author; (2) year of publication; (3) country of enrollment; (4) ethnicity of the study population; (5) age and gender of individuals included; (6) diagnostic criteria for IDD cases; (7) genotyping methods; (8) source of controls; (9) matching items; (10) number of subjects under IDD cases and controls; (11) Relation with IDD; Data were extracted carefully from all eligible publications independently by two investigators. For conflict resolution, an agreement was reached by discussion.

### Methodological quality assessment

The two investigators assessed the qualities of all the included studies separately using the Clark scores system, which contains 10 items [24, 25]. Scores below 5 indicate low quality, while 5–7 scores represent moderate quality and 8–10 scores denote high quality [24, 25].

### Statistical analysis

The PRISMA checklists and their guidelines were cautiously followed during the whole process of the study [26]. The association strength between *COL9A3* trp3 polymorphism and IDD risk was assessed by combining ORs with 95%CI. The estimations of pooled ORs were determined by the weighted average OR from each study. Significance was identified by a *P*-value less than 0.05 in Z-test. The pooled ORs and 95%CI were calculated for trp3 positive (the mutation type) versus trp3 negative (the wild type). Because seven studies [11, 14, 18, 23, 27–29] included in this meta-analysis only exhibited data in “Trp3 positive versus Trp3 negative” form. In other words, these studies did not have enough data to calculate ORs and 95%CI for five comparison models. In addition, although the other four studies [17, 21, 22, 30] included this meta-analysis showed separate data in homozygous type (TGG/TGG), heterozygous type (TGG/CGG) and wild type (the others which not include TGG at this site, such as CGG/CGG) for trp3, the number of subjects for homozygous type (TGG/TGG) was too small with no more than two subjects observed in each study. So we combined homozygous and heterozygous type together as trp3 positive (TGG/TGG, TGG/CGG) and the wild type was defined as trp3 negative (the others which not include TGG at this site). In other words, the trp3 positive was defined as the presence of at least 1 Trp3 allele and the trp3 negative were the types without Trp3 allele. The statistical heterogeneity was verified by *I*^*2*^ statistics. Fixed-effect model was used to estimate the ORs and 95%CI when heterogeneity was low (*I*^*2*^ < 50%), while the random effects was adopted when heterogeneity was high (*I*^*2*^ > 50%) [31]. Sensitivity analyses were also performed to evaluate the function of an individual study on the pooled ORs by removing each study in turn. All analyses were performed using STATA 14 (Stata, College Station, TX). Subgroup analyses were performed to find whether sex or ethnicity of studies was linked to the value of the pooled ORs and 95%CI as well. Because only few studies included separate data of degree of IDD, we do not conduct a subgroup analysis stratified by disease degree. All *P*-values were two-sided. Publication bias was checked using the Begg funnel plot [32] and the Egger’s test [33] (*P* < 0.05 was considered statistically significant).

## Results

### Characteristics of the studies

**Fig. 1:**
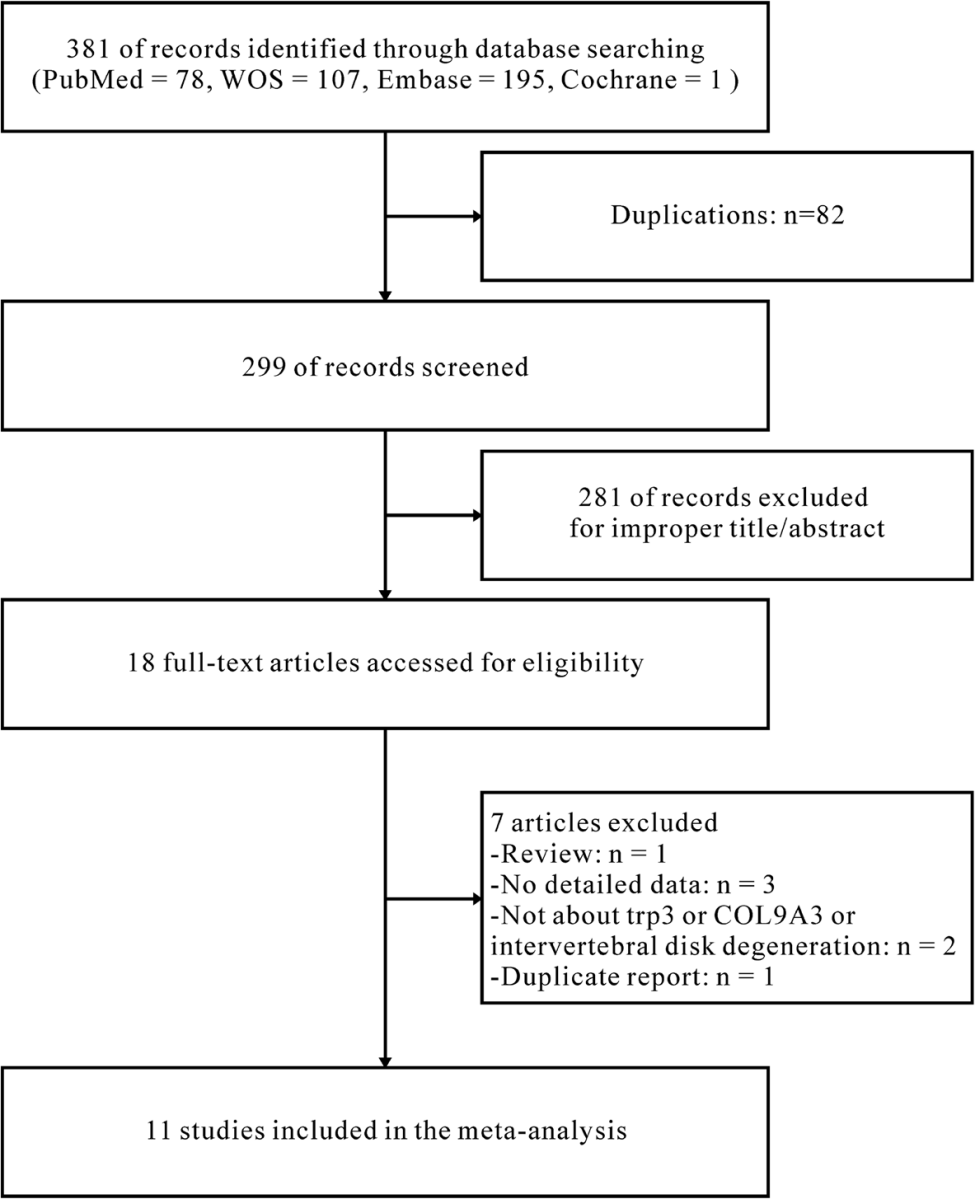
Flow diagram for the selection of studies

A flow chart, presented as Fig. 1, describes the exclusion/inclusion of publications. The comprehensive articles search screened out 381 potentially relevant articles, of which 82 articles were excluded for duplication and 281 articles were removed because of obvious irrelevance after browsing the title and/or abstract. Two articles were excluded because they did not study trp3, *COL9A3* or IDD; one article was removed because it is a duplicate report; three articles were excluded because they did not have detailed data; another article was removed because it was a review. Finally, 11 case-control studies [11, 14, 17, 18, 21–23, 27–30] were identified due to the inclusion criteria.

As shown in Tables 1, 11 eligible studies for *COL9A3* trp3 with 1631 cases of IDD and 1366 controls were included in this meta-analysis. The characteristics of all the included studies are also listed in the Tables 1 and 2, including the year, country and continent of studies, the ethnicity, age and gender of subjects, the diagnosis methods, genotyping methods, source of controls, matching items of cases and controls, relation with IDD and the number of subjects in control/case group in each studies. The quality assessment of study was listed in Table 3.

**Table 1:** Main Characteristics of Studies Included in This Meta-analysis for *COL9A3* trp3 Polymorphisms

Study ID	year	Enrolled Country	Ethnicity	Age	Gender	Diagnosis by	Genotyping Method	Control Source	Matching	Cases	Controls	Relation with IDD
Bagheri et al. [18]	2016	Iran	Iranian	20~ 66	both	MRI	PCR-seq	individuals with acute trauma and patients without IDD	gender	108	57	only present in male subgroup
Solovieva et al. [14]	2006	Finland	N/D	40~ 45	men	MRI	PCR-seq	Patients without IDD	HWE, age	77	55	Absent
Kales et al. [27]	2004	Greece	Southern European	< 60	both	surgery, MRI or CT	PCR-seq	patients without IDD and visitors to nonsurgical, nonorthopedic units	BMI	105	102	Absent
Paassita et al. [17]	2001	Finland	Finnish	< 78	N/D	both clinic and MRI or CT	PCR-seq CSGE	healthy individuals, osteoarthritis, rheumatoid arthritis, chondrodysplasias	N/D	171	321	Present
Kelempisioti et al. [30]	2011	Finland	N/D	young adult, mean age 21	both	MRI	PCR-seq	Patients without IDD	gender, HWE	292	246	Absent
Matsui et al. [28]	2004	USA	most of them are Caucasian	16~ 87	both	N/D	PCR-seq	Individuals with a vertebral fracture treated by fusion	N/D^a^	97	10	Absent^b^
Eskola et al. [21]	2010	Finland	Caucasian	12 ~ 14	both	MRI	PCR-seq	children without IDD	age, weight, BMI, HWE	66	154	Absent
Rathod et al. [22]	2012	India	Indian	15~ 60	both	both clinic and MRI	PCR-seq and TaqMan assay	Patients without IDD	age	100	100	Absent
Zhu et al. [29]	2011	USA	most of them are Caucasian	16~ 78	both	N/D	N/D	Individuals with a vertebral fracture treated by fusion	age	26	6	N/D
jim et al. [23]	2005	China	Chinese	18~ 55	both	MRI	PCR-seq CSGE	Patients without IDD	age, gender	514	290	Absent
Toktas et al. [11]	2015	Turkey	N/D	35~ 45	male	MRI	PCR-seq	healthy individuals	age, gender	75	25	Absent^c^

Abbreviations: *PCR-seq* restriction analysis polymerase chain reaction sequencing, *N/D* not described, *CSGE* conformation sensitive gel electrophoresis, *MRI* magnetic resonance imaging, *CT* Computed Tomography, *IDD* intervertebral disk degeneration, *HWE* Hardy–Weinberg equilibrium, *BMI* body mass index. ^a^This study matched age, gender, race between trp3 positive and trp3 negative groups, but not between control and case groups. ^b^, although absent relation with IDD, case group was positively associated with the diagnosis of spinal stenosis with spondylolisthesis. ^c^, although absent relation with IDD, control group has significantly higher scores in Pfirrmann classification than case group

**Table 2:** Distribution of Genotypes and *COL9A3* trp3 Polymorphisms Among Cases and Controls

Study ID	Year	Continent	Gender	Case		Control	
				Trp3 positive	Trp3 negative	Trp3 positive	Trp3 negative
Bagheri et al. [18]	2016	Asia	male	12	21	2	21
			female	17	58	8	26
			overall	29	79	10	47
Solovieva et al. [14]	2006	Europe	male	15	62	8	47
Kales et al. [27]	2004	Europe	male	6	62	1	46
			female	3	34	4	51
			overall	9	96	5	97
Paassita et al. [17]	2001	Europe	unknown	40	131	30	291
Kelempisioti et al. [30]	2011	Europe	overall	43	249	52	194
Matsui et al. [28]	2004	America	overall	7	90	0	10
Eskola et al. [21]	2010	Europe	male	2	28	14	59
			female	7	29	17	64
			overall	9	57	31	123
Rathod et al. [22]	2012	Asia	male	3	62	7	60
			female	2	33	0	33
			overall	5	95	7	93
Zhu et al. [29]	2011	America	male	3	11	0	2
			female	3	9	0	4
			overall	6	20	0	6
Jim et al. [23]	2005	Asia	overall	0	290	0	514
Toktas et al. [11]	2015	Europe	male	5	70	0	25

**Table 3:** Quality Assessment of the Included Articles

Author	Year	A	B	C	D	E	F	G	H	I	J	Sum
Bagheri et al. [18]	2016	1	0	1	1	1	0	0	1	1	0	6
Solovieva et al. [14]	2006	1	1	1	1	1	0	0	1	1	0	7
Kales et al. [27]	2004	1	0	1	1	1	0	0	1	1	0	6
Paassita et al. [17]	2001	1	0	1	1	1	0	0	1	0	0	5
Kelempisioti et al. [30]	2011	0	1	1	1	1	0	0	1	1	0	6
Matsui et al. [28]	2004	0	0	1	1	1	1	0	1	0	0	5
Eskola et al. [21]	2010	1	1	1	1	1	0	0	1	1	0	7
Rathod et al. [22]	2012	1	0	1	1	1	0	0	1	1	0	6
Zhu et al. [29]	2011	0	0	1	1	1	0	0	1	1	0	5
Jim et al. [23]	2005	1	0	1	1	1	0	0	1	1	0	6
Toktas et al. [11]	2015	0	0	1	1	1	0	0	1	1	0	5

Abbreviations: *A* Control group, *B* Hardy–Weinberg equilibrium, *C* Case group, *D* Primer, *E* Reproducibility, *F* Blinding, *G* Power calculation, *H* Statistics, *I* Corrected statistics, *J* Independent replication, *Sum* sum of quality assessment score, *1* done, *0* undone or unclear

However, Jim et al. [23] measured the 804 subjects with no Trp3 positive subjects neither in case nor in control groups.(Table 2) This proportion is largely deviated from other studies included. We speculate that it might be something wrong in its genotyping method or there might be a selection bias in its subjects. So Jim et al. [23] is excluded in the following analyses.

### Association between *COL9A3* trp3 polymorphism and IDD risk in overall

**Fig. 2:**
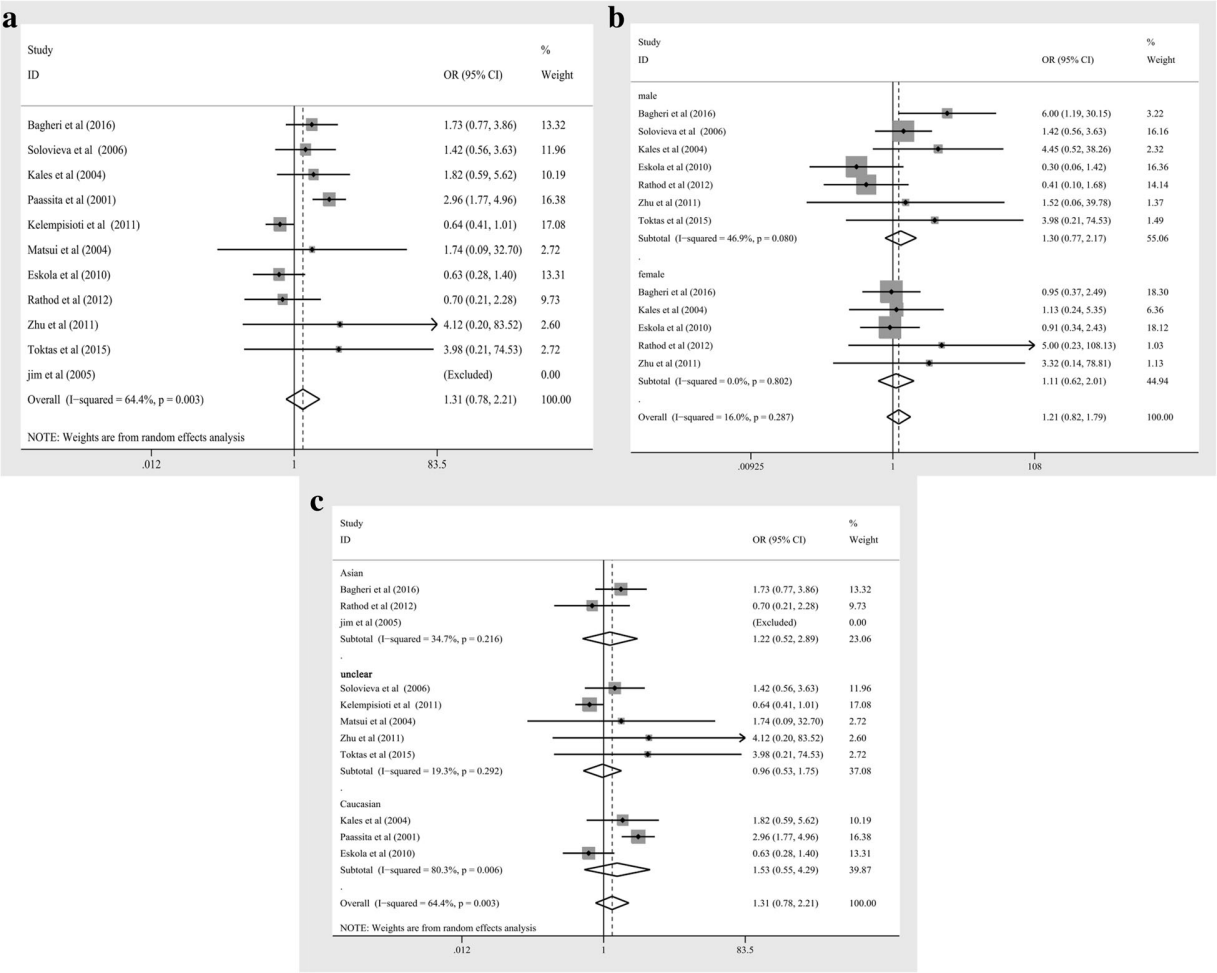
Forest plot of the association between *COL9A3* trp3 polymorphisms and IDD (trp3 positive versus trp3 negative) for: (**a**) overall analysis and subgroup analyses stratified by (**b**) gender, (**c**) ethnicity

Significant heterogeneity was found among the studies of trp3 in the overall meta-analysis. So the random effects model was applied to evaluate the connection between trp3 polymorphism and IDD risk. The result of the evaluation showed that there was no association of trp3 polymorphism with IDD risk (as shown in Fig. 2a and Table 4, trp3 positive versus trp3 negative: ORs = 1.31, 95%CI = 0.78–2.21, *P* = 0.309; heterogeneity test χ^2^ = 25.31, *P* < 0.10, *I*^2^ = 64.40%).

**Table 4:** Statistics of polled ORs and Heterogeneity for Overall and Sub Group Analyses of *COL9A3* trp3 Polymorphism

*COL9A3* trp3		*N*	ORs analyses		Heterogeneity Analyses			Model Used for Meta-analysis
			polled ORs (95% CI)	*P* value	χ^2^	P_heterogeneity_	I^2^ (%)	
Overall		11	1.31 (0.78,2.21)	0.309	25.31	0.003	64.40%	Random
sub group analysis by gender	male	7	1.30 (0.77,2.17)	0.322	11.30	0.080	46.90%	Fixed
	female	5	1.11 (0.62,2.01)	0.725	1.64	0.802	0.00%	
sub group analysis by ethnicity	Asian	3	1.22 (0.52,2.89)	0.645	1.53	0.216	34.70%	Random
	Caucasian	3	1.53 (0.55, 4.29)	0.417	10.17	0.006	80.3%	
	unclear	5	0.96 (0.53, 1.75)	0.907	4.96	0.292	19.3%	

Abbreviations: *CI* confidence interval, *ORs* odds ratios, *N* number of studies included in each analysis

### Subgroup analysis between *COL9A3* trp3 polymorphism and IDD risk based on gender

Subgroup meta-analysis of the studies based on gender (male and female) showed no significant heterogeneity. Thus, the fixed effects model was used to assess the relationship between trp3 polymorphism and IDD risk. The result of the evaluation indicated that trp3 polymorphism was not associated with IDD risk in both gender (as shown in Fig. 2b and Table 4, for male subgroup, trp3 positive versus trp3 negative: ORs = 1.30, 95%CI = 0.77–2.17, *P* = 0.322; heterogeneity test χ^2^ = 11.30, *P* < 0.10, *I*^2^ = 46.90%; for female subgroup, trp3 positive versus trp3 negative: ORs = 1.11, 95%CI = 0.62–2.01, *P* = 0.725; heterogeneity test χ^2^ = 1,64, *P* > 0.10, *I*^2^ = 0.00%).

### Subgroup analysis between *COL9A3* trp3 polymorphism and IDD risk based on ethnicity

Subgroup analysis was conducted according to different ethnicity (Asian, Caucasian and unclear) and was observed significant heterogeneity. So the random effects model was used to test the association between trp3 polymorphism and IDD risk. The result of the calculations indicated that trp3 polymorphism had no associations to IDD risk in any ethnicity (as shown in Fig. 2c and Table 4, for Asian subgroup, trp3 positive versus trp3 negative: ORs = 1.22, 95%CI = 0.52–2.89, *P* = 0.645; heterogeneity test χ2 = 1.53, *P* > 0.10, *I*^2^ = 34.70%. for Caucasian subgroup, trp3 positive versus trp3 negative: ORs = 1.53, 95%CI = 0.55–4.29, *P* = 0.417; heterogeneity test χ^2^ = 10.17, *P* < 0.10, *I*^2^ = 80.30%; for unclear subgroup, trp3 positive versus trp3 negative: ORs = 0.96, 95%CI = 0.53–1.75, *P* = 0.907; heterogeneity test χ^2^ = 4.96, *P* > 0.10, *I*^2^ = 19.3%).

### Sensitivity analysis

**Fig. 3:**
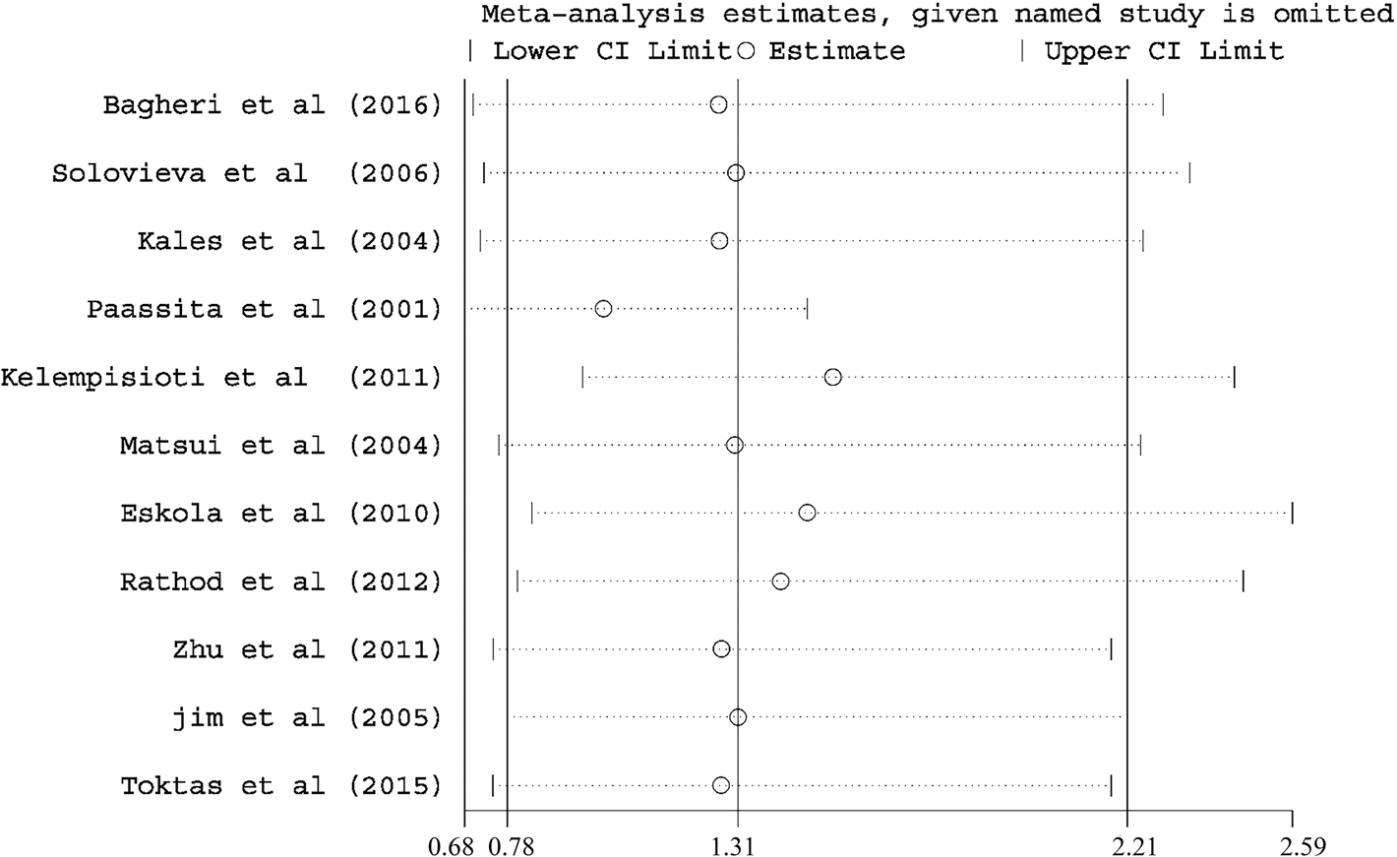
Sensitivity analysis for confirmation of the stability of the pooled results

Sensitivity analysis was conducted to evaluate the influence set by the individual study on the pooled ORs for *COL9A3* trp3 polymorphism by deleting one study each turn in every genetic model (as shown in Fig. 3). There was no change in the significance of any outcomes, indicating the stability of the results in this meta-analysis.

### Publication Bias

**Fig. 4:**
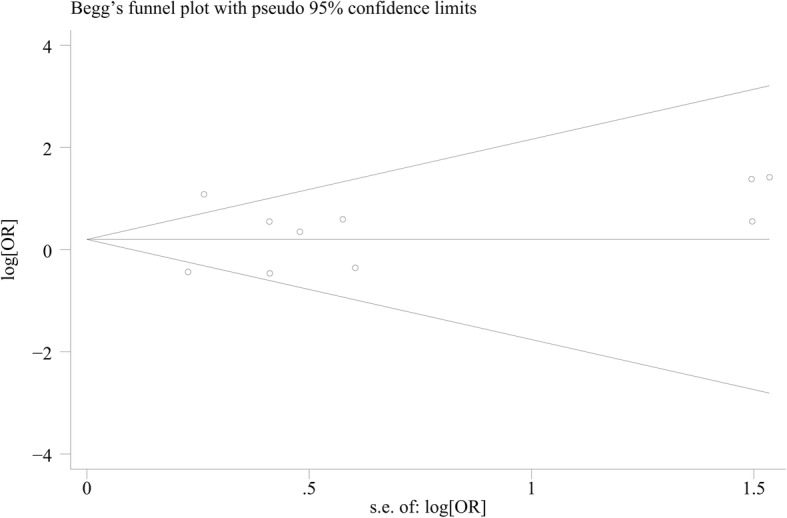
Begg funnel plot for the identification of potential missing studies

The Begg funnel plot (as shown in Fig. 4) and the Egger’s test were performed to assess publication bias in the selected literature. No evidence of publication bias was observed in this study (Begg’s test: *P* = 0.283, Egger’s test: *t* = 0.54, 95%CI = − 2.88–4.64, *P* = 0.606 for *COL9A3* trp3).

## Discussion

IDD, a common musculoskeletal disease, is widely considered as multifactorial diseases enforcing economic and medical burdens to society. Genetic factors have been considered as one of the leading causes of IDD [7, 11, 34]. *COL9A3*, an extracellular matrix molecule present in the nucleus pulposus of the intervertebral disc and cartilage, codes for Collagen IX [35]. Collagen IX is vital for the normal cartilage development or maintenance. Mutations in *COL9A3* could cause chondrodysplasias in humans as well as articular cartilage and intervertebral discs degeneration in mice [36]. *COL9A3* gene was observed to be a key genetic influencer in the process of IDD [23]. Previous studies have reported the association between the *COL9A3* trp3 polymorphism and IDD, but with conflicting results. With the studies with larger sample sizes of predisposing gene polymorphism, it would be much more reliable to discover the connection between candidate genes and specific type of diseases. In order to solve the inconsistence, meta-analysis was performed to examine the association of *COL9A3* trp3 polymorphism with IDD risk by critically reviewing 11 studies. Its strength came from the accumulation of various published data, offering more information to explore significant differences.

The pooled ORs (trp3 positive versus trp3 negative) and 95%CI did not show a significant association of *COL9A3* trp3 polymorphism with IDD risk in the overall populations. Different *COL9A3* trp3 frequencies have been reported in two genders of subjects (male and female) [18]. So we performed a stratified analysis by gender to determine whether there was an association between trp3 and IDD differed by gender. We also found no association of *COL9A3* trp3 polymorphism with IDD risk both in male or female subgroup. To our limited knowledge, ethnicity may contribute to different genetic characteristics of IDD. Hence, we also performed a subgroup analysis by ethnicity and the outcomes indicated no association of *COL9A3* trp3 polymorphism with IDD risk in any of ethnicity subgroup. Based on the analyses above, we speculated that *COL9A3* trp3 might be a minor factor in genetic etiology of IDD risk due to the small amount of *COL9A3* inside the intervertebral discs [37]. All of the results above do not eliminate the possibility of a clinically vital association that remains to be explored more carefully in convincing studies of larger sample sizes.

No significant heterogeneity was observed in subgroup analysis of gender, whereas there existed heterogeneities in the overall comparisons and subgroup analysis of continent for trp3 and IDD risk. To search the source of heterogeneity, we observed that *I*^2^ values had significantly decreased after excluding Paassita et al. [17] or Kelempisioti et al. [30] in overall analysis. We also found that *I*^2^ values had significantly decreased after excluding a study of Paassita et al. [17] in subgroup analysis of Europe continent. The results indicated that the major source of the heterogeneity might result from these studies. However, heterogeneity did not seem to influence the results, because the lack of association between trp3 and IDD was not altered after excluding either of study mentioned above.

Moreover, no significant change of results was identified by sensitivity analyses, which indicating the reliability of results. These suggested the reliability of the results. Publication bias was also tested in this study. On the basis that a meta-analysis collects various data from numerous studies, the effect of publication bias among the articles included in the study can influence the meta-analytic results. Neither the Egger’s test nor the Begg funnel plot showed significant publication bias for this analysis. Although the results are reliable, more studies are required to be conducted in order to confirm the outcome of this meta-analysis.

Our meta-analysis has several strengths. Firstly, to our best knowledge, this is the first meta-analysis focusing on the connection between *COL9A3* trp3 polymorphism and the risk of IDD. We suggest that such a method of incorporating the outcomes of related studies may help us to understand the effect of polymorphism on disease development better. Secondly, we also have taken the gender and ethnicity of subjects into account. This study included researches of Asia (Iran, China and India), Europe (Finland, Greece and Turkey) and America (USA), containing different kinds of ethnicities and enrolling both male and female. So the results are much more comprehensive. Moreover, several strategies and strict principle were applied to evaluate the methodological quality of the studies and most of the studies included in this meta-analysis possessed moderate or high qualities.

The present meta-analysis also has a few limitations that should be taken into account. Firstly, the number of filtered studies for *COL9A3* trp3 polymorphism is a little bit small. Secondly, the heterogeneity was a little bit high when overall and sub group of ethnicity analyses were conducted, contributing to a cautious acceptance of the results. What’s more, some studies were removed from our research for lacking detailed data, which may contribute to selection bias. Limited to data, we only analyze trp3 positive versus trp3 negative to estimate the ORs and 95%CI rather than five models (allele, homozygote, recessive, dominant and heterozygote models). This may also influence the reliability of outcomes. Finally, although most of articles in this meta-analysis made a good match of age, gender or other items which might influence the results, some articles did not take certain items into account or even did not mention the match points. These confounding factors might affect the results.

## Conclusions

Basing on the epidemiological evidence, our meta-analysis suggested that *COL9A3* trp3 polymorphism did not seem to be connected to risk of IDD in any gender, continent or ethnicity of people. Future researches with larger sample sizes are required to verify this outcome.

## Abbreviations

CI: Confidence interval
*COL9A3*: Collagen type IX alpha 3 chain
IDD: Intervertebral disk degeneration
LBP: Low back pain
ORs: Odds ratios
WOS: Web of Science
